# Trends in Deficiency Citations in Florida Assisted Living Facilities

**DOI:** 10.1093/geroni/igaa057.283

**Published:** 2020-12-16

**Authors:** Hari Sharma

**Affiliations:** The University of Iowa, Iowa City, Iowa, United States

## Abstract

Despite numerous anecdotal reports of poor quality and residential safety concerns in Assisted Living Facilities (ALFs), there is limited federal oversight of ALFs. Usually, state surveyors conduct inspections of ALFs for compliance with regulations and issue deficiency citations and/or fine non-compliant facilities. Florida is one of the few states that publicly releases inspections data. The aim of this study is to fill the gap in our understanding of ALF quality by examining the trends in deficiency citations in Florida. We obtain data on 1,047 ALFs with 25 or more beds operating in Florida between 2012-2018. We use descriptive methods to examine the trends in citations over time and further stratify by profit status. We also evaluate whether facilities get cited for the same deficiencies repeatedly. Every year, approximately, one third of the facilities were free of any deficiency citations. From 2012 to 2018, fewer facilities were cited for resident care and medication but more facilities were cited for training and staffing. Approximately 45.8% of not-for-profit and 35.1% of for-profit facilities were free of deficiency citations in 2018. A majority of facilities cited for a given deficiency were cited at least once again for that deficiency within the study period. Florida ALFs appear to be improving only in some deficiencies but getting worse in some other deficiencies. Furthermore, repeat citations are common suggesting that facilities fail to improve their care/service patterns to avoid repeat citations. More stringent regulations and stricter enforcements may deter facilities from repeat citations.

